# Recurring nodular scleritis following inactivated vaccination for COVID‐19

**DOI:** 10.1002/ccr3.7420

**Published:** 2023-06-13

**Authors:** Alireza Peyman, Mohsen Pourazizi, Shakiba Dehghani, Sarah Ghorbani

**Affiliations:** ^1^ Department of Ophthalmology, Isfahan Eye Research Center Isfahan University of Medical Sciences Isfahan Iran; ^2^ Department of Ophthalmology, Farabi Eye Hospital Tehran University of Medical Science Tehran Iran

**Keywords:** coronavirus disease‐2019, COVID‐19 vaccine, scleritis, Sinopharm

## Abstract

**Key Clinical Message:**

Though COVID‐19 vaccination saved many lives all around the world, it has had many adverse effects including ophthalmologic side effects. It is important to report such adverse effects to provide timely diagnosis and management.

**Abstract:**

Since the COVID‐19 global outbreak, many types of vaccines have been introduced. These vaccines have been associated with some adverse effects including ocular manifestations. Herein, we report a case of a patient who developed nodular scleritis shortly after receiving the first and second doses of the inactivated COVID‐19 vaccine, Sinopharm.

## 1 INTRODUCTION

Since the beginning of the COVID‐19 pandemic known as severe acute respiratory syndrome coronavirus 2 (SARSCoV2) which is caused by the novel coronavirus, different types of vaccines including protein subunit, inactivated, mRNA, and vector‐based have been approved against this virus.^1^, ^2^

Several inflammatory adverse effects and immune‐related conditions, including ocular adverse effects associated with COVID‐19 vaccination, have been reported.^3^, ^4^

In this report, we describe a case of nodular scleritis following inactivated COVID‐19 vaccine, Sinopharm (China National Pharmaceutical Group Co., Ltd.).

## 2 CASE PRESENTATION

**FIGURE 1:**
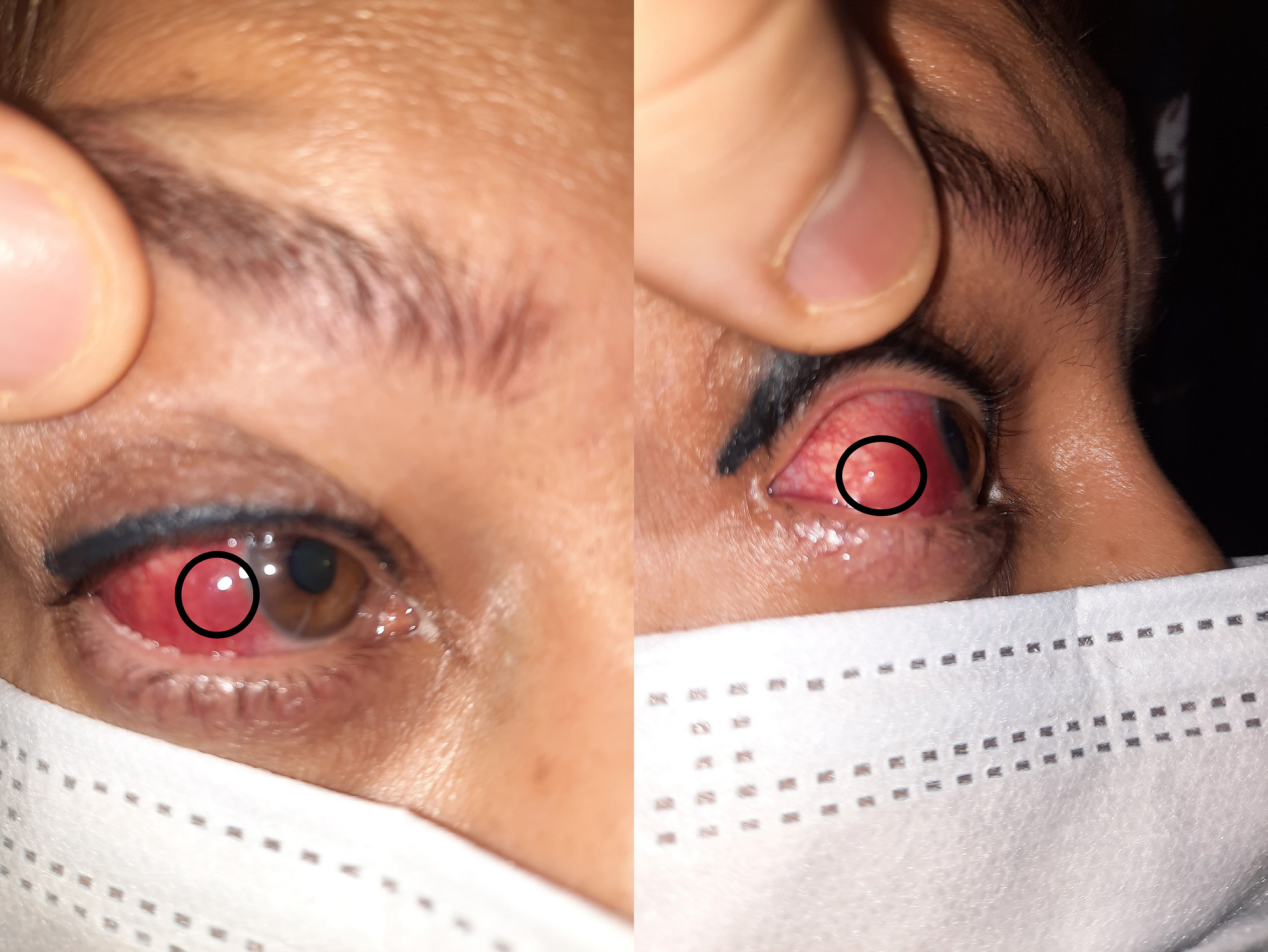
External examination of the patient's right eye following first dose of the inactivated COVID‐19 vaccination, revealed purplish scleral hyperemia, and a nodule on the temporal sclera.

A 53‐year‐old woman was referred to the referral outpatient clinic of ophthalmology affiliated with the Isfahan University of medical sciences, Isfahan, Iran, in October 2021, with severe ocular pain, redness, and photophobia in her right eye. Her symptoms started 10 days after receiving the first dose of the inactivated COVID‐19 vaccine, Sinopharm (China National Pharmaceutical Group Co., Ltd.). Her past medical history was unremarkable and she did not have any suspected symptoms of COVID‐19 during the past 2 years. On physical examination, her uncorrected visual acuity (UCVA) was 20/25 OD and 20/30 OS and her best‐corrected visual acuity (BCVA) was 20/20 in both eyes. Pupils and ocular motility were normal. External examination of her right eye revealed purplish scleral hyperemia and a nodule on the temporal sclera (Figure 1). There was no corneal or anterior chamber inflammation. Intraocular pressure (IOP) was 16 mm Hg and fundus examination was normal.

Based on the clinical findings and paraclinical investigation, she was diagnosed with nodular scleritis. As so, treatment with topical 1% prednisolone eye drops 4 times a day, oral non‐steroidal anti‐inflammatory drug (NSAID) indomethacin 25 mg every 8 h and Pantoprazole 40 mg daily was started. After significant improvement in ocular symptoms and signs for a week, steroid eye drops and oral NSAID were tapered.

A rheumatologic consult was done to find out any systemic disease as the underlying cause of nodular scleritis. Complete systemic investigation including complete blood count, erythrocyte sedimentation rate, C‐reactive protein, serum antibody screening (ANA, anti‐DNA antibody, rheumatic factor, antineutrophil cytoplasmic antibody), serum uric acid, syphilis serologic test, urinalysis, and chest X‐ray was done and found to be within normal limits.

**FIGURE 2:**
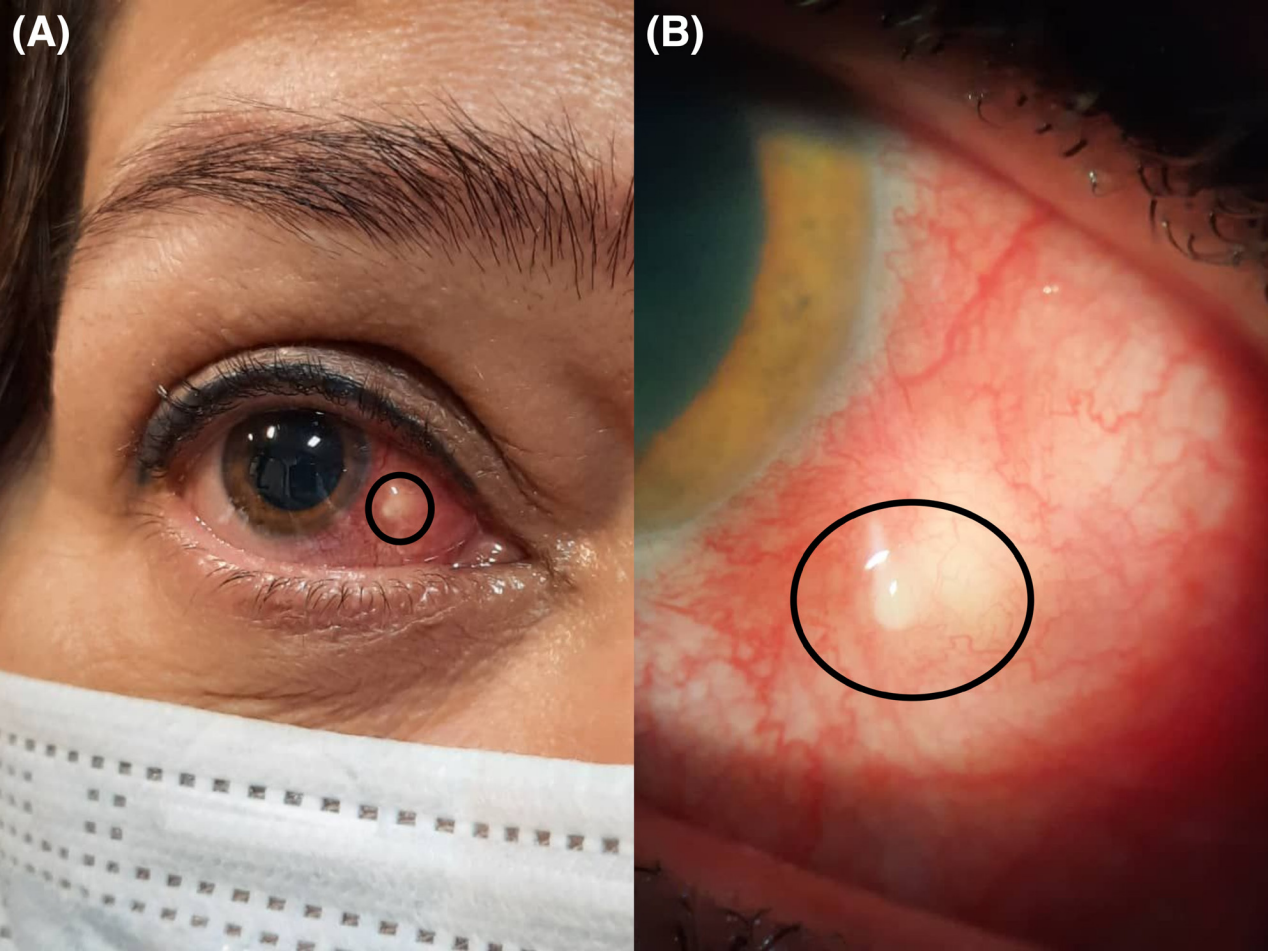
External examination (A) and slit‐lamp examination (B) showed violaceous scleral hyperemia which did not blanch on instillation of phenylephrine and a nodular lesion on the nasal sclera of the patient's right eye after receiving second dose of the inactivated COVID‐19 vaccine.

In January 2022, 4 days after receiving the second dose of Sinopharm vaccine (China National Pharmaceutical Group Co., Ltd.), she developed severe ocular pain and redness in the same eye for the second time. Her BCVA was 20/20 in both eyes. Slit‐lamp examination showed violaceous scleral hyperemia which did not blanch on instillation of phenylephrine and a nodular lesion on the nasal sclera of her right eye (Figure 2). No abnormality was seen in the anterior chamber, IOP was 15 mm Hg and fundus examination was unremarkable. Anterior segment optical coherences tomography showed thickening of the nasal sclera of her right eye compared to the left eye. Systemic evaluations did not show any remarkable positive findings.

A causality assessment was performed using the Naranjo Adverse Drug Reaction Probability Scale. Our patient's total Naranjo Scale score was 9 (definite adverse drug reaction) (Table 1).^5^ Then, the diagnosis of recurrent nodular scleritis was made. The patient was started on oral NSAID and steroid eye drops to control recurrent episodes. After 2 weeks of treatment initiation, ocular signs, and symptoms markedly improved.

**TABLE 1:** The Naranjo algorithm for adverse reaction for a patient with nodular scleritis associated with COVID‐19 vaccination. Naranjo Adverse Drug Reaction Probability Scale was 9 as a definite adverse drug reaction.

Question	Yes	No	Do not know	Score
1. Are there previous conclusive reports on this reaction?	+1	0	0	+1
2. Did the adverse event appear after the suspected drug was administered?	+2	−1	0	+2
3. Did the adverse event improve when the drug was discontinued or a specific antagonist was administered?	+1	0	0	0 (do not know)
4. Did the adverse event reappear when the drug was readministered?	+2	−1	0	+2
5. Are there alternative causes that could on their own have caused the reaction?	‐1	+2	0	+2
6. Did the reaction reappear when a placebo was given?	‐1	+1	0	0 (do not know)
7. Was the drug detected in blood or other fluids in concentrations known to be toxic?	+1	0	0	0 (do not know)
8. Was the reaction more severe when the dose was increased or less severe when the dose was decreased?	+1	0	0	0 (do not know)
9. Did the patient have a similar reaction to the same or similar drugs in any previous exposure?	+1	0	0	+1
10. Was the adverse event confirmed by any objective evidence?	+1	0	0	+1
	Total score: 9			

## 3 DISCUSSION

The case presented in this study illustrates nodular scleritis as a possible side effect of inactivated COVID‐19 vaccine (Sinopharm).

Scleritis is a rare and vision‐threatening autoimmune disorder which can involve either the anterior or posterior sclera. Scleritis has a wide spectrum of clinical presentations which include severe ocular pain, redness, and globe tenderness.^6^ Nearly half of the patients with scleritis have an underlying systemic disease.^7^ However, in our patient, there was no evidence of infection or autoimmune disease.

Overall, the prevalence of adverse effects of Sinopharm vaccine is lower compared to *Pfizer*‐BioNTech COVID‐19 vaccine and AstraZeneca COVID‐19 vaccine.^8^

Several ocular complications including anterior uveitis, acute macular neuroretinopathy, and maculopathy, central serous retinopathy, acute abducens nerve palsy, orbital myositis, and inflammatory pseudotumor following COVID‐19 vaccination have been reported.^9^, ^10^, ^11^, ^12^, ^13^ Orbital inflammatory pseudotumor was diagnosed in a woman presented with blepharoptosis following COVID‐19 vaccination. Imaging showed left lacrimal gland enlargement and diffuse enlargement of the left lateral and superior rectus muscle.^13^

Although the exact mechanisms of COVID‐19 vaccines associated ocular adverse events are not yet completely known, the possible mechanisms of these adverse effects include molecular mimicry, antibody‐mediated hypersensitivity reactions, and inflammatory damage induced by certain vaccine adjuvants.^3^

Though being rare, development of scleral inflammation after administration of vaccine has been reported in few articles.^14^, ^15^, ^16^

Most of the reported cases, were middle‐aged to old women who developed symptoms ranging from ocular redness and pain to visual impairment within 10 days of vaccination and they were all successfully treated with corticosteroids.

In a multinational case series by Testi et al.,^3^ seven cases of scleritis after COVID‐19 vaccination were reported. These cases were following Pfizer, Astra‐Zeneca and Moderna vaccines' administration and only in one of the cases, the condition recured after receiving second dose of the vaccine.

Pichi et al.^14^ reported case series of seven patients with ocular manifestations after inactivated COVID‐19 vaccination. One of these cases was a patient with a medical history of rheumatoid arthritis who presented with redness and pain in both eyes, 10 days after receiving inactivated COVID‐19 vaccine. The diagnosis of diffuse scleritis in this patient was made based on clinical findings and the patient was successfully treated with topical steroids.

In another report, Younus et al. reported case of a 78‐year‐old woman presented with new onset headache, severe retro orbital pain, and visual impairment after receiving the second dose of the ChAdOx1‐S vaccine (Oxford/AstraZeneca). She was then diagnosed with posterior scleritis and successfully treated with oral prednisolone.^15^

Peripheral ulcerative keratitis (PUK) and nodular scleritis following inactivated COVID‐19 vaccination, was reported by Penbe.^16^ A 76‐year‐old man was admitted with severe ocular pain and vision loss 2 weeks after inactivated COVID‐19 vaccination. The patient undergone advanced treatment for PUK including oral steroids, azathioprine, topical cyclosporine with topical dexamethasone, corneal amniotic membrane grafting, and penetrant keratoplasty.^16^

Following inactivated COVID‐19 vaccination, Pang et al. reported case of nodular scleritis in a 78‐year‐old man which presented with lacrimation, redness, and visual loss.^17^ He experienced mild symptoms and was responsive to glucocorticoids.

Savino et al.^18^ presented a case of unilateral scleritis and ocular myositis and another case of bilateral scleritis after mRNA BNT162b2 COVID‐19 vaccine (Pfizer/BioNTech) administration. Unilateral scleritis and ocular myositis were diagnosed in a 64‐year‐old woman who had received COVID‐19 vaccine 5 days before and presented with preorbital swelling, redness, pain, limitation in movement, and proptosis. She responded completely to oral prednisolone. Bilateral redness and painful eye movement were the presenting symptoms of a 58‐year‐old woman who had received vaccine and was then diagnosed with bilateral scleritis and undergone treatment with topical dexamethasone that resulted in complete and rapid resolution of symptoms.

In our case study, relapsing nodular scleritis after the second dose of COVID‐19 vaccination in a patient without any underlying condition, strongly suggests a relationship between administration of the vaccine and onset of the disease. However, whether this event is coincidental or associated with vaccine administration, cannot be fully established and further studies are required.

## AUTHOR CONTRIBUTIONS

**Alireza Peyman:** Conceptualization; data curation; investigation; resources; supervision; validation; writing – review and editing. **Mohsen Pourazizi:** Conceptualization; data curation; methodology; project administration; resources; supervision; writing – review and editing. **Shakiba Dehghani:** Formal analysis; investigation; methodology; resources; software; writing – original draft. **Sarah Ghorbani:** Conceptualization; formal analysis; investigation; software; validation; writing – original draft.

## FUNDING INFORMATION

None of the authors has any financial disclosures.

## CONFLICT OF INTEREST STATEMENT

The authors declare that there is no conflict of interests regarding the publication of this paper.

## ETHICS STATEMENT

Ethics approval for this report was obtained from the Ethics Committee of Isfahan University of Medical Sciences, Isfahan, Iran. A signed statement of informed consent to publish patient data obtained from the patient.

## Data Availability

The data that support the findings of this study are available from the corresponding author upon reasonable request.
